# A medium hyperglycosylated podocalyxin enables noninvasive and quantitative detection of tumorigenic human pluripotent stem cells

**DOI:** 10.1038/srep04069

**Published:** 2014-02-12

**Authors:** Hiroaki Tateno, Yasuko Onuma, Yuzuru Ito, Keiko Hiemori, Yasuhiko Aiki, Madoka Shimizu, Kumiko Higuchi, Masakazu Fukuda, Masaki Warashina, Susumu Honda, Makoto Asashima, Jun Hirabayashi

**Affiliations:** 1 Research Center for Stem Cell Engineering, National Institute of Advanced Industrial Science and Technology (AIST), Tsukuba Central 2, 1-1-1 Umezono, Tsukuba, Ibaraki 305-8568, Japan; 2 Research Center for Stem Cell Engineering, National Institute of Advanced Industrial Science and Technology (AIST), Tsukuba Central 4, 1-1-1 Higashi, Tsukuba, Ibaraki 305-8562, Japan; 3 Cell Biology Research Center, Life Science Research Laboratories, Wako Pure Chemical Industries, Ltd. 6-1 Takada-cho, Amagasaki, Hyogo 661-0963, Japan

## Abstract

While human pluripotent stem cells are attractive sources for cell-replacement therapies, a major concern remains regarding their tumorigenic potential. Thus, safety assessment of human pluripotent stem cell-based products in terms of tumorigenicity is critical. Previously we have identified a pluripotent stem cell-specific lectin probe rBC2LCN recognizing hyperglycosylated podocalyxin as a cell surface ligand. Here we demonstrate that hyperglycosylated podocalyxin is secreted from human pluripotent stem cells into cell culture supernatants. We establish a sandwich assay system, named the GlycoStem test, targeting the soluble hyperglycosylated podocalyxin using rBC2LCN. The GlycoStem test is sufficiently sensitive and quantitative to detect residual human pluripotent stem cells. This work provides a proof of concept for the noninvasive and quantitative detection of tumorigenic human pluripotent stem cells using cell culture supernatants. The developed method should increase the safety of human pluripotent stem cell-based cell therapies.

Human pluripotent stem cells (hPSCs), such as embryonic stem cells (hESCs) and induced pluripotent stem cells (hiPSCs) are attractive sources for cell replacement therapies due to their properties of self-renewal and pluripotency^1^,^2^. Extensive research has been conducted with these cells to produce various cell types. Several pluripotent stem cell-based therapeutics entered clinical trials. In 2012, clinical trials have been conducted with retinal pigment epithelial (RPE) cells derived from hESCs to treat patients with dry age-related macular degeneration and Stargart's macular dystrophy^3^. However, stem cell-based therapies clearly bring with them new safety challenges. The most obvious safety risk is tumorigenicity of residual undifferentiated cells^4^,^5^,^6^. To minimize patient risk, each stage of the cell therapy production should be assessed for potential safety concerns prior to introduction of the cells into a patient^5^. The properties of a cell must therefore be characterized by evaluating various markers of undifferentiated, differentiated, and undesired cells. Evaluation of such markers has been performed using conventional assays, such as flow cytometry, immunohistochemistry, and quantitative real-time PCR (qRT-PCR), used singly and in combination^7^. Alternatively, an *in vivo* teratoma formation assay using severe combined immunodeficiency (SCID) mice provides a straightforward means to assess the existence of tumorigenic stem cells in a cell population. However, all of these currently available methods necessitate the use of a significant number (>10^4^) of invaluable cells. Thus, “continuous monitoring” of the cells during the cell manufacturing process, i.e., from undifferentiated to differentiated states, is impractical.

Previously, we performed comprehensive glycome analysis of a large set of hiPSCs (114 cell types) and hESCs (9 cell types) using a high-density lectin microarray^8^ and found that a lectin designated rBC2LCN (recombinant N-terminal domain of BC2L-C), identified from *Burkholderia cenocepacia*, binds exclusively to all of the undifferentiated hPSCs tested, but not to the differentiated somatic cells^8^. In a practical sense, rBC2LCN has served as a useful probe for both staining and sorting of hPSCs^9^. Recently, podocalyxin, a hyperglycosylated sialomucin, was identified as a predominant cell surface ligand of rBC2LCN^10^. rBC2LCN exhibited significant affinity to a mucin-type *O*-glycan comprising an H type3 structure prepared from human 201B7 iPSCs^10^,^11^. Therefore, it was suggested that H type3 is a novel hPSC marker recognized by rBC2LCN^10^. A small single-chain protein (16 kDa), rBC2LCN can be expressed at high levels in a soluble form in *Escherichia coli* (>80 mg/L) and easily purified to homogeneity by one-step sugar-immobilized affinity chromatography. In contrast, the antibody is a large protein (>140 kDa) composed of two subunits (heavy and light chains) that requires mammalian cells to produce. Thus, rBC2LCN has high potential to serve as a novel type of detection reagent targeting extensive hPSCs, particularly given its cost-effectiveness and high productivity.

Here we show that hyperglycosylated podocalyxin recognized by rBC2LCN is secreted from hPSCs into cell culture supernatants. The rBC2LCN-captured podocalyxin was detected with another lectin probe rABA, that recognizes high density mucin-type *O*-glycans on podocalyxin. A major advantage of the developed sandwich assay system is that it requires cell culture supernatants, but not cells, for quantification of residual tumorigenic hPSCs, so that invaluable cells used for transplantation are not consumed. This work provides a novel concept for the use of cell culture supernatants for the safety assessments of stem cell-based products.

## Results

### rBC2LCN binds to cell culture supernatants of hiPSCs

We have previously demonstrated that rBC2LCN binds to undifferentiated hiPSCs and hESCs, but not to differentiated somatic cells^8^,^9^. Also, podocalyxin was identified as a predominant glycoprotein ligand of rBC2LCN on hiPSCs and hESCs^10^. Here we first examined the possibility that cell culture supernatants, instead of cells, could be used for discrimination between differentiated and undifferentiated cells by rBC2LCN. For this purpose, cell culture supernatants of hiPSCs derived from TIG3 hiPSC#19 (TIG3 hiPSC#19 sup) or control cell culture media (Control media, see *Methods*) were directly labeled with Cy3 and incubated overnight at 20°C with rBC2LCN immobilized on a glass slide. After washing, binding was detected using an evanescent-field activated fluorescence scanner. As shown in Fig. S1, cell culture supernatants of TIG3 hiPSCs (TIG/MKOS #19) gave much higher signals than control media only. This finding implies that the pluripotent state of stem cells may be analyzed using cell culture supernatants.

### GlycoStem test

**Figure 1:**
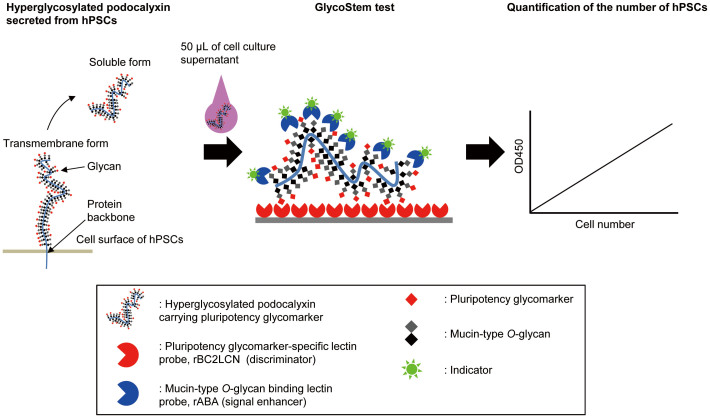
Schematic representation of the principle of the GlycoStem test. Hyperglycosylated podocalyxin, a type1 transmembrane protein, carries a hiPSC/hESC marker (H type3, Fucα1-2Galβ1-3GalNAc) recognized by the hiPSC/hESC-specific lectin probe rBC2LCN (discriminator). Podocalyxin (soluble form) is secreted into cell culture supernatants, and is captured by rBC2LCN immobilized on a microtiter plate. The rBC2LCN-captured podocalyxin is detected with HRP-labeled rABA (signal enhancer) recognizing mucin-type *O*-glycans heavily displayed on podocalyxin. The number of hPSCs is quantified with the GlycoStem test using cell culture supernatants.

We then attempted to establish a practical system to measure rBC2LCN-positive media. For this purpose, it seemed reasonable to adopt a sandwich assay system to enhance the significant but relatively weak signals. Assuming hyperglylcosyated podocalyxin as a major target molecule^10^, selection of an overlay probe which works best as a “signal enhancer” is critical, (Fig. 1). Cell culture supernatants of TIG3 hiPSCs (TIG/MKOS #19) or control media were incubated with rBC2LCN immobilized on a glass slide. After washing, Cy3-labeled overlay-probe candidates were incubated at 20°C for 3 h, and their binding was analyzed by an evanescence-field fluorescence scanner^12^. We first tried goat anti-podocalyxin polyclonal antibody (pAb) as an overlay probe toward a conventional antibody-lectin sandwich assay system^13^,^14^, but without success, although the antibody could be used for western blotting and immunoprecipitation of podocalyxin^10^. We then challenged a novel system of “lectin-lectin” sandwich assay, which utilizes two glycan-specific probes (Fig. 1). As a result of screening 44 recombinant lectins to enhance the rBC2LCN signals, 17 were shown to give higher signals to TIG3 hiPSCs (TIG/MKOS #19) supernatants than control cell culture media (Fig. S2). Four recombinant lectins, *Sclerotium rolfsii* lectin (rSRL), *Coprinopsis cinerea* lectin 2 (rCGL2), *Agaricus bisporus* lectin (rABA), and *Xerocomus chrysenteron* (rXCL) exhibited strong enough signals (>10,000) to cell culture supernatants of TIG3 hiPSCs (TIG/MKOS #19), while giving only little or no signal to control media (<2,500). This result demonstrates that the four lectins could serve as strong signal enhancers. For the subsequent studies, rABA was used as an overlay molecule, which gave the best S/N ratio in the ELISA-type assay described below.

**Figure 2:**
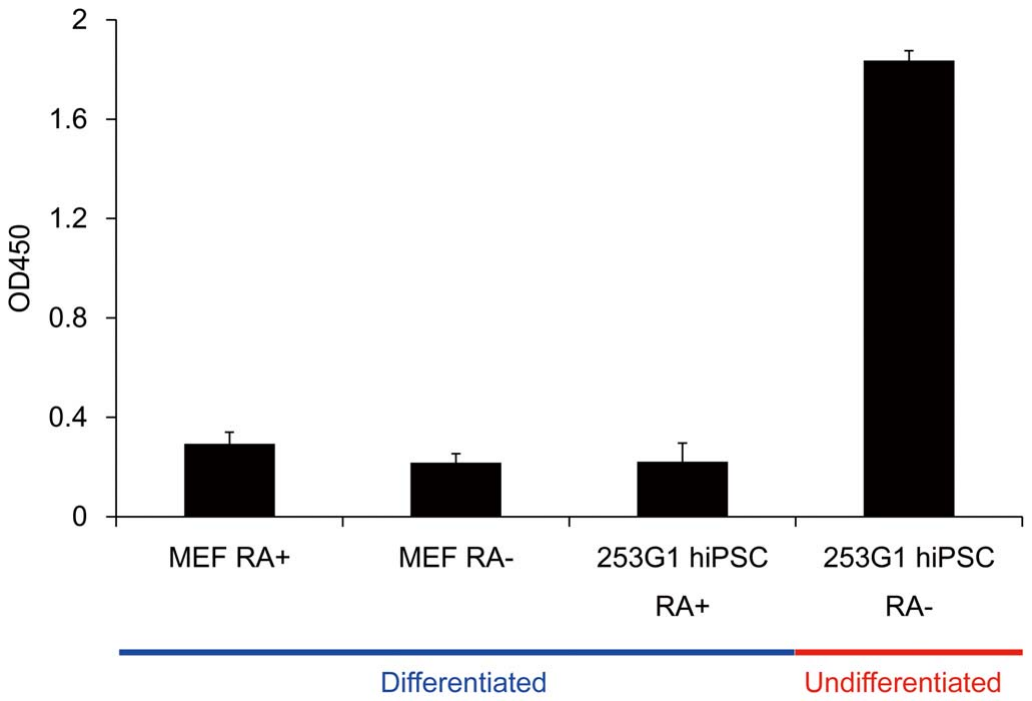
The GlycoStem test discriminates undifferentiated cells from differentiated cells. Biotinylated rBC2LCN (0.1 μg/well) was immobilized on streptavidin-coated 96-well microtiter plates at 37°C for 1 h. Cell culture supernatants of MEF and 253G1 hiPSCs with or without retinoic acid (RA) treatments for 15 days were incubated at 37°C for 1 h. After washing, HRP-labeled rABA (0.1 μg/mL, 50 μL) was overlayed at 37°C for 1 h. After washing, absorbance at 450 nm was then detected. Absorbance at 450 nm of the control cell culture media was subtracted from the values obtained from the cell culture supernatants. Data are shown as mean ± SD of triplicate samples.

Since no such lectin-lectin sandwich assay system has ever been reported, we decided to establish such a system using an ELISA (enzyme-linked immunosorbent assay)-type 96-well microtiter plate. Biotinylated rBC2LCN (0.1 μg/well) was immobilized on a streptavidin-coated 96-well microtiter plate at 37°C for 1 h. After cell culture supernatants of 253G1 hiPSCs were incubated at 37°C for 1 h, horseradish peroxidase (HRP)-labeled recombinant lectins (0.1 μg/mL, 50 μL) were overlaid at 37°C for 1 h and the absorbance at 450 nm was measured (for details, see *Methods*). Mouse feeder cells with (MEF RA+) or without (MEF RA-) 15-day retinoic acid treatment, and differentiated 253G1 hiPSCs with 15-day retinoic acid treatment (253G1 hiPSC RA+) were also analyzed for comparison (Fig. 2). As a result, in the developed rBC2LCN-rABA sandwich assay, much-enhanced signals were observed for undifferentiated 253G1 hiPSCs without retinoic acid treatment (253G1 hiPSC RA-, OD450 = 1.8), while they were almost at basal levels for differentiated cells of MEF RA+ (OD450 = 0.3), MEF RA- (OD450 = 0.2), and 253G1 hiPSC RA+ (OD450 = 0.2). These results demonstrate unequivocally that the developed system (designated the GlycoStem test) discriminates undifferentiated hiPSCs from differentiated cells using cell culture supernatants rather than precious cells.

### Evidence that podocalyxin is a soluble ligand captured by rBC2LCN

**Figure 3:**
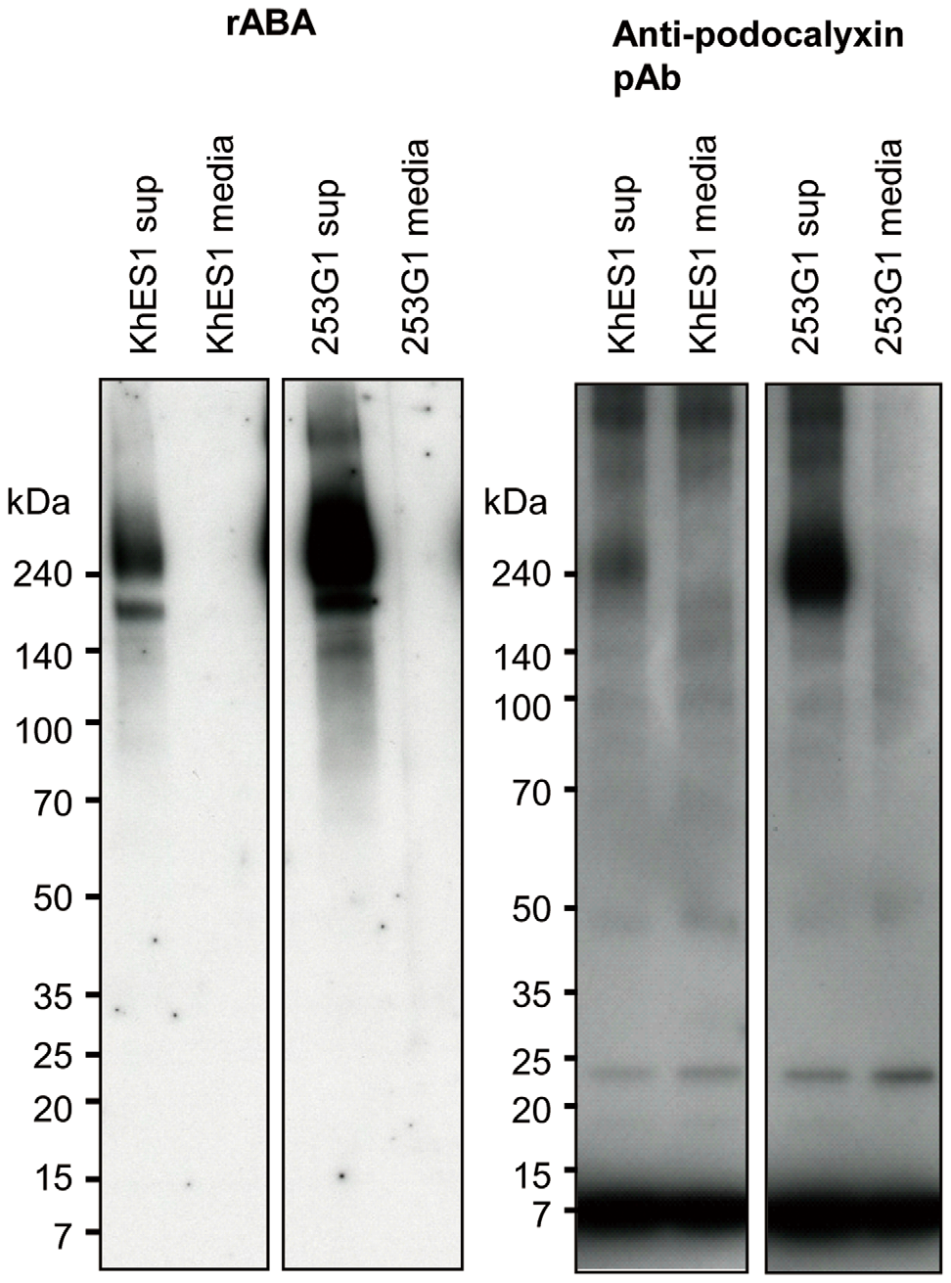
Podocalyxin is a soluble ligand of the GlycoStem test. Cell culture supernatants of KhES1 hESCs (KhES1 sup), 253G1 hiPSCs (253G1 sup), and the corresponding control cell culture media (KhES1 media and 253G1 media) were incubated with 10 μL of rBC2LCN-coated magnetic beads. After washing, bound samples were eluted, electrophoresed under reducing conditions, and blotted with 0.1 μg/mL of HRP-labeled rABA (left panel) or 0.1 μg/mL of goat anti-podocalyxin pAb (R&D) followed by HRP-labeled donkey anti-goat IgG (right panel).

Having developed the GlycoStem test, we searched for its target ligands secreted from hiPSCs and hESCs. Cell culture supernatants (100 μL) of hESCs (KhES1 sup), hiPSCs (253G1 sup), and the corresponding control cell culture media (KhES1 media and 253G1 media) were incubated with 10 μL of rBC2LCN-coated magnetic beads. After washing the beads, bound samples were eluted, electrophoresed under reducing conditions, and blotted with HRP-labeled rABA. As shown in Fig. 3, a major band was detected at >240 kDa in both hESCs and hiPSCs and a weaker band between 140 and 240 kDa. We have previously demonstrated that podocalyxin, which has a high molecular mass of >240 kDa, is a cell-surface ligand of rBC2LCN on hiPSCs and hESCs^10^, implying that the observed >240 kDa protein might be soluble podocalyxin. As expected, this >240 kDa band was stained with goat anti-podocalyxin pAb in both hiPSCs and hESCs in blotting experiments, where protein is denatured, although anti-podocalyxin pAb failed to be used as an overlay probe for intact form of soluble podocalyxin in the GlycoStem test (Fig. 3). These results clearly demonstrate that the target secreted glycoprotein ligand in the GlycoStem test is podocalyxin (Fig. 1).

### Standard curve

**Figure 4:**
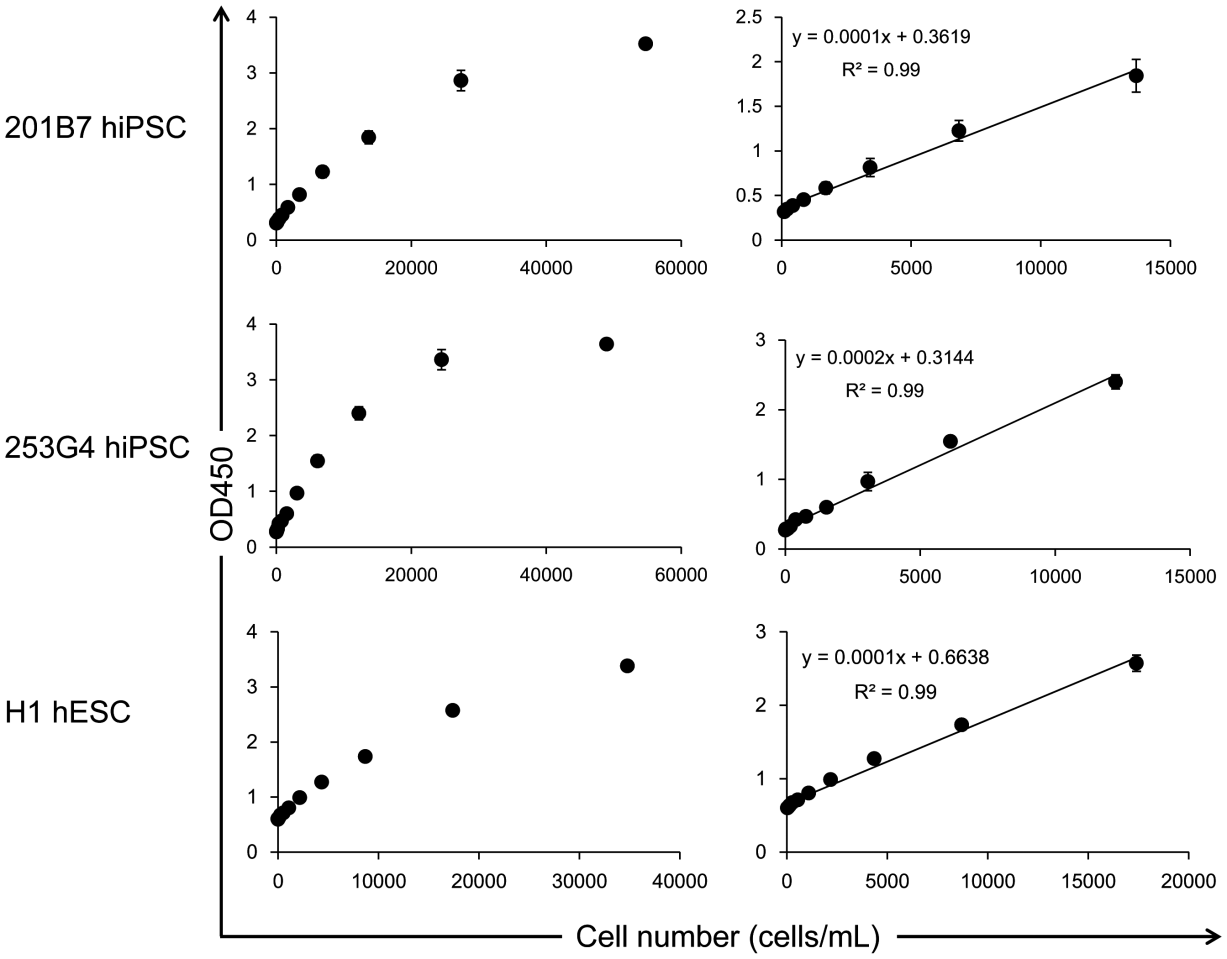
Standard curve. Cell culture supernatants of 201B7 hiPSCs (upper panel) and 253G4 hiPSCs (middle panel) cultured in StemSure hPSC medium, and H1 hESCs (lower panel) cultured in mTeSR1 were recovered and serially diluted with the corresponding culture media, while the adhered cells were recovered and counted. The obtained cell culture supernatants were analyzed by the GlycoStem test in triplicates. The absorbance at 450 nm of the media only was subtracted from the values obtained from the cell culture supernatants. Data are shown as mean ± SD of three independent experiments.

It is critical to develop a quantitative assay to estimate the cell number of hPSCs using cell culture supernatants. Thus, we generated standard curves for hiPSCs (201B7 and 253G4) and hESCs (H1). These cells were cultured in either 2.5 mL of StemSure hPSC medium (hiPSCs) or 2 mL of mTeSR1 (hESCs) for 24 h, following the cell culture supernatants were recovered, serially diluted with the corresponding cell culture media, and analyzed by the GlycoStem test, while the adhered cells were recovered and counted. As a negative control, the assay was carried out using the control media only. The absorbance at 450 nm of the control media was subtracted from the values obtained from the cell culture supernatants. As shown in Fig. 4, signals were obtained in a concentration-dependent manner. A linear regression revealed the linear range of detection (R^2^ > 0.98). We also found that the GlycoStem test could be applied to various other cell culture media including Nutristem (Fig. S3), ReproFF (Fig. S4), MEF-conditioned medium (MEF-CM) (Fig. S5), and mTeSR1 (Fig. S6). The linear range of detection with R^2^ > 0.98 was obtained for all of these defined media.

### Lower limit of detection (LLOD) of the GlycoStem test

**Figure 5:**
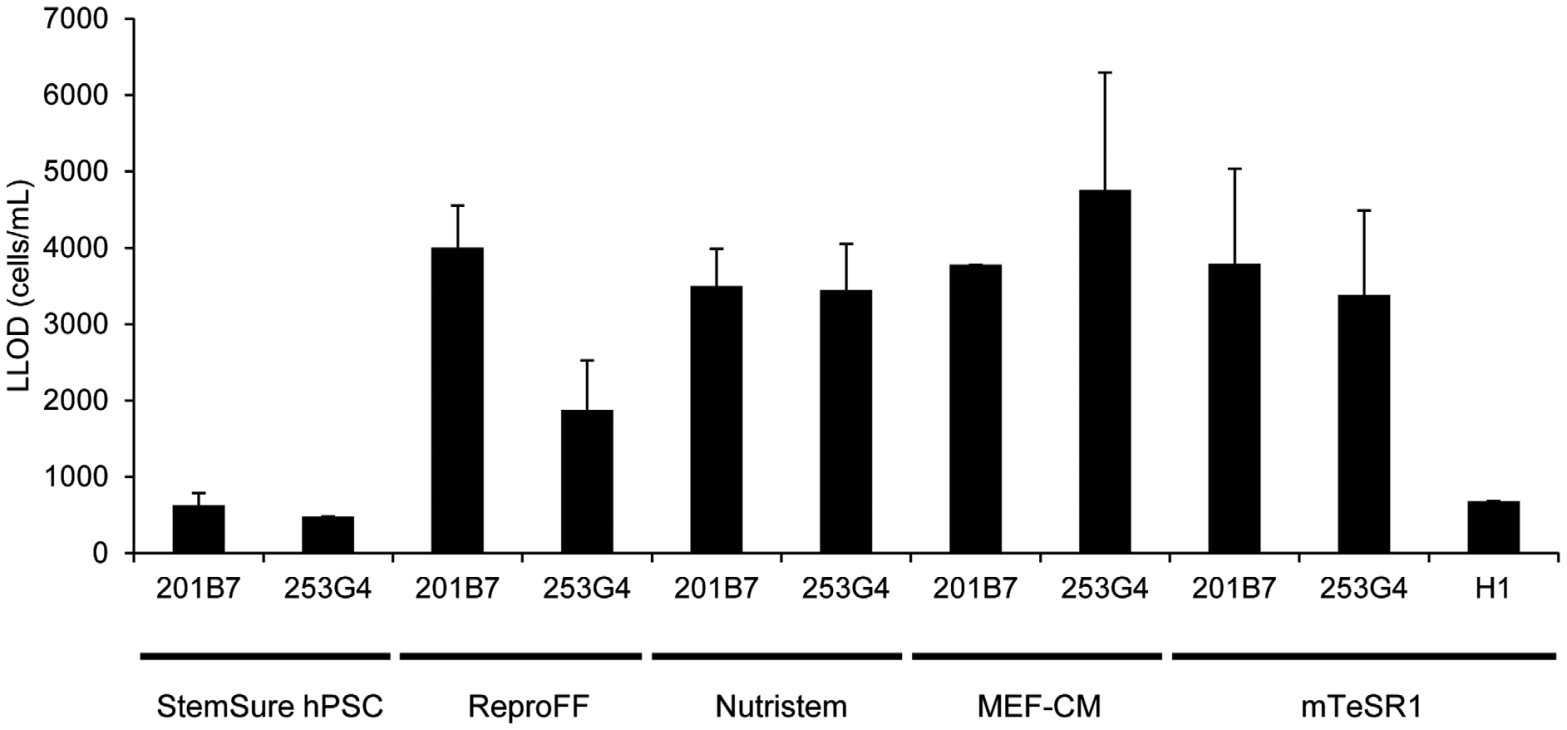
Lower limit of detection (LLOD) of the GlycoStem test. The LLOD of the signal was calculated as the mean plus 3.3-fold the standard deviation of the measurement of the negative control media. Data are shown as mean ± SD of three independent experiments.

We compared the lower limit of detection (LLOD) for 201B7, 253G4, and W01 cells cultured in various types of cell culture media in the developed GlycoStem test. The value was calculated for each medium and cell type as the mean plus 3.3-fold the standard deviation of the measurement of the negative control medium (Fig. 5)^7^. The LLOD values varied largely depending on the types of culture media, while the variations between cell types (i.e., 201B7 and 253G4) were relatively small (Fig. 5). The lowest LLOD was obtained for StemSure hPSC medium (Ave LLOD = 623 cells/mL for 201B7 and 478 cells/mL for 253G4), while other media were also applicable to this system with somewhat higher LLOD values (680–4,753 cells/mL). It should be noted that hiPSCs and hESCs cultured in various cell culture media were positive for both anti-SSEA4 (Fig. S7) and rBC2LCN (Fig. S8), indicating that the different LLOD values are not due to the contamination of differentiated cells.

### Detection of hiPSCs in mixed cell cultures

**Figure 6:**
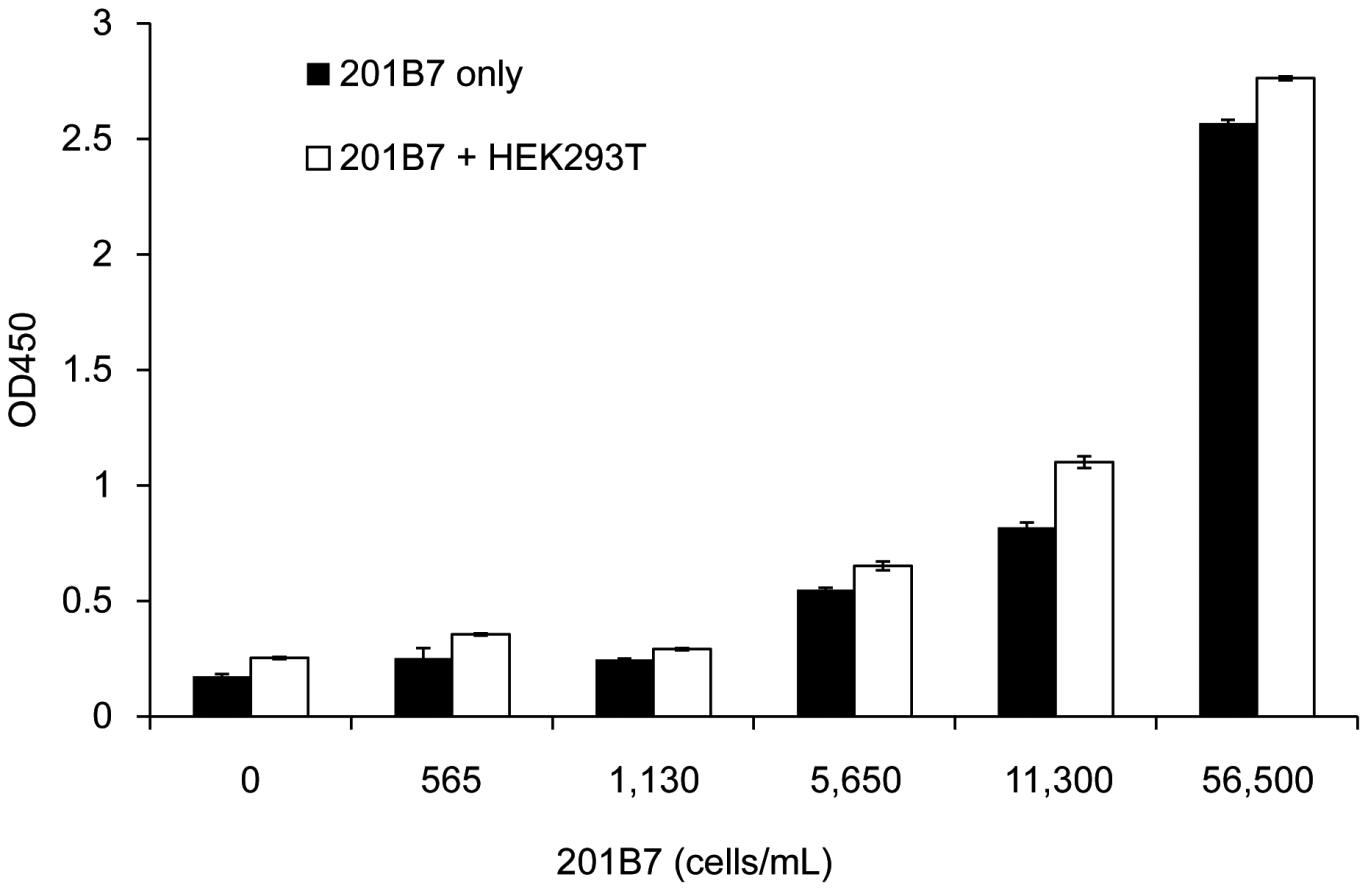
Detection of hiPSCs in a mixed cell culture. 201B7 hiPSCs (2.2 × 10^3^–2.3 × 10^5^) were cultured either in the presence (white box) or absence (black box) of HEK293T cells (1.39 × 10^6^ cells) in 2 mL of mTeSR1 in a 6 well plate. Cells were recovered and counted, while cell culture supernatants were analyzed by the GlycoStem test. The absorbance at 450 nm of the buffer only was subtracted from the values obtained for the cell culture supernatants. Data are shown as mean ± SD of triplicates.

We then assessed whether the system can be used to detect hiPSCs in a mixed cell culture. 201B7 hiPSCs (2.2 × 10^3^ – 2.3 × 10^5^) were cultured either in the presence or absence of HEK293T cells (1.39 × 10^6^ cells) in 2 mL of mTeSR1 in a 6 well plate. Cells were recovered and counted, while cell culture supernatants were analyzed by the GlycoStem test. As shown in Fig. 6, 201B7 hiPSCs gave signals, either in the presence (white box) or absence (black box) of HEK293T cells, in a cell number-dependent manner. No or little effect on the presence of HEK293T cells was observed. 201B7 cells (5,650 cells/mL) could be sufficiently detected even in the presence of HEK293T cells, which is similar to the LLOD values obtained for 201B7 cultured in mTeSR1 (3,792 cells/mL, Fig. 5). These results demonstrate that hiPSCs in a mixed cell cultures could be detected by the GlycoStem test.

### Monitoring the state of pluripotency during differentiation

**Figure 7:**
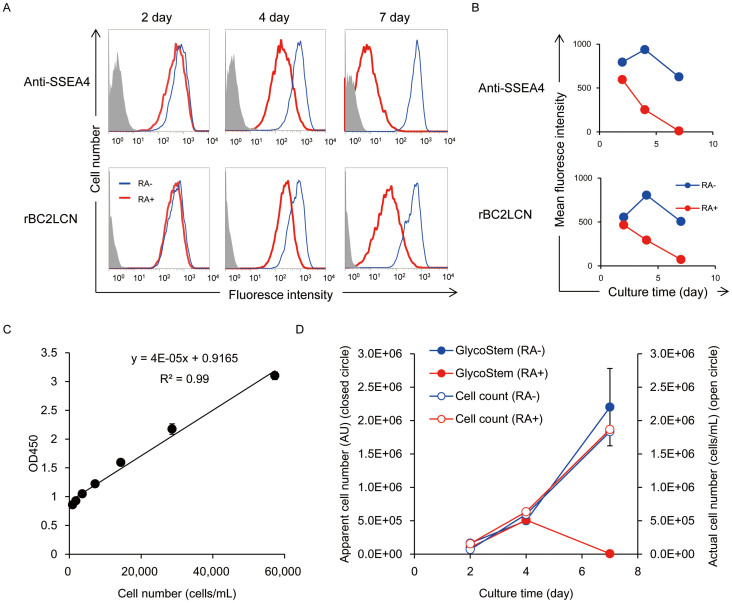
Monitoring the state of pluripotency during differentiation. 201B7 hiPSCs were cultured in mTeSR1 in the presence (red line) or absence (blue line) of 10 μM retinoic acid. After 2, 4, and 7 days, cells were stained with anti-SSEA4 (clone MC-813-70, 1:300 dilution, Millipore) followed by AlexaFluor488-labeled anti-mouse IgG(Molecular Probes)(A, upper panel). Cells were also stained with HiLyteFluor 647-labeled rBC2LCN (A, lower panel). Cells were then analyzed by FACS Aria. Grey, negative control. (B) Mean fluorescence intensities of the flow cytometry data. (C) Standard curve of the GlycoStem test generated using cell culture supernatants of 201B7 hiPSCs cultured for 24 h. Data are shown as mean ± SD of triplicate samples. (D) The GlycoStem test. Data of the GlycoStem test are shown in mean ± SD of triplicate samples. Similar results were obtained in three independent experiments. The apparent cell number was calculated from the linear equation [the apparent cell number = (OD450 − 0.9165)/4 × 10^−5^] obtained from the standard curve and expressed as “AU” (closed circle). The actual total cell number is expressed as “cells/mL” (open circle).

Finally, we applied the GlycoStem test to monitor the state of pluripotency during differentiation. 201B7 hiPSCs were cultured in a 6-well plate in 2 mL of mTeSR1 in the presence or absence of 10 μM retinoic acid. After 2, 4, and 7 days, cells were recovered, counted, and stained with anti-SSEA4 and rBC2LCN, while cell culture supernatants were analyzed by the GlycoStem test (Fig. 7). In the GlycoStem test, a standard curve was generated using cell culture supernatants of 201B7 hiPSCs cultured in mTeSR1 for 24 h without retinoic acid (Fig. 7C). The apparent cell number was then calculated from the linear equation obtained from the standard curve: the apparent cell number = (OD450 − 0.9165)/4 × 10^−5^. The cell number estimated by the GlycoStem test was expressed as an “arbitrary unit (AU)”. In the absence of retinoic acid, the number of hiPSCs estimated with the GlycoStem test increased with culture time (Fig. 7D, blue closed circles), similarly to the actual cell count (Fig. 7D, blue open circles). In the presence of retinoic acid, anti-SSEA4 and rBC2LCN staining gradually decreased with culture time and were almost absent on day 7 in the flow cytometer analysis (Figs. 7A and 7B, red). The apparent cell number of hiPSCs was also estimated by the GlycoStem test (Fig. 7D). On day 4, the cell number of hiPSCs estimated by the GlycoStem test was 5.1 × 10^5^ AU (Fig. 7D, red closed circles), while the actual cell count was 6.3 × 10^5^ cells/mL (Fig. 7D, red open circles). On day 7, the value obtained by the GlycoStem test was greatly decreased (6,208 AU, Fig. 7D, red closed circles), while the actual cell count was increased (1.9 × 10^6^ cells/mL, Fig. 7D, red open circles), indicating that cells are mostly differentiated. These results demonstrate that the GlycoStem test is capable of monitoring the state of pluripotency during differentiation in a quantitative and noninvasive manner.

## Discussion

To minimize patient risk, each stage of the stem cell-derived therapeutic development must be rigorously assessed for potential safety concerns. In particular, differentiation state should be tracked closely along with the development process, since a major concern with stem cell therapy is that residual undifferentiated cells could form tumors in the patient^5^. With this aspect in mind, Kuroda et al recently evaluated three conventional methods to detect residual undifferentiated hiPSCs; soft agar colony formation, flow cytometry, and quantitative RT-PCR^7^. They concluded that quantitative RT-PCR using Lin28 as a target gene was the most sensitive and rapid assay, which could detect 0.002% residual undifferentiated hiPSCs in RPE cells induced from hiPSCs (i.e., a single hiPSC in 5 × 10^4^ RPE cells) within 6 h, while the LLOD values determined for soft agar colony formation and flow cytometry were estimated to be 1 (500 hiPSCs in 5 × 10^4^ RPE cells) and 0.1% (50 hiPSCs in 5 × 10^4^ RPE cells), respectively. However, all of these conventional methods consume a significant amount of invaluable cells (>10^4^ cells) for the analysis. This makes it basically difficult to perform continuous monitoring of the state of differentiation. Here we developed, for the first time, a rapid, sensitive and quantitative method to diagnose hPSCs in a noninvasive manner; i.e., using only a small volume (50 μL) of cell culture supernatant. The GlycoStem test is quantitative so that the system should contribute to the safety assessment of stem cell-based products.

In the GlycoStem test, a standard curve was generated using cell culture supernatants of 201B7 hiPSCs cultured in mTeSR1 for 24 h. The cell number estimated by the GlycoStem test was expressed as AU. The obtained values by the GlycoStem test could vary depending on the degree of undifferentiation/differentiation of hPSCs. However, this doesn't mean that the values obtained by the system are inaccurate, but the values rather provide “apparent cell number”, which also includes another parameter such as cell conditions. For standardization, standard compounds without lot-to-lot differences are demanded. We are now challenging to prepare such standard materials.

The sensitivities, in terms of LLOD, varied substantially depending on the types of cell culture media used. Among them, StemSure hPSC medium gave the lowest LLOD (478 cells/mL) consistent with the lowest background, while other media were also applicable to this system, but with somewhat higher LLOD values (680–4,753 cells/mL). This suggests that the GlycoStem test might detect >0.05% (500 cells) of undifferentiated cells, if 10^7^ of transplanting differentiated cells are cultured in 10 mL of cell culture media. Thus, the GlycoStem test should be sufficiently sensitive to apply some types of hPSC-derived therapeutic cells. It should be assessed the applicability of the developed system for hPSC-derived therapeutic cells such as RPE cells and cardiomyocytes. To apply the system for a wide variety of hPSC-derived cells, we are now challenging to improve the sensitivity of the system. Furthermore, it is important to obtain the direct relationship between the values obtained by the GlycoStem test and the teratoma-forming ability. This could provide the threshold for teratoma-forming ability using the values obtained by the GlycoStem test.

Forty-four recombinant lectins were screened for an overlay probe candidate to establish the sandwich assay. Among them, four recombinant lectins - rSRL, rCGL2, rABA, and rXCL - performed best as signal enhancers. Interestingly, rSRL, rABA, and rXCL belong to the same lectin family (FB_lectin, PF07367), all of which exhibit specificity to Galβ1-3GlcNAc (core 1), a typical mucin-type *O*-glycan, suggesting that the rBC2LCN-captured ligands are *O*-glycosylated. Consistently, the rBC2LCN-captured glycoprotein ligand was found to be hyperglycosylated podocalyxin, which is equipped with a mucin domain as well as five potential *N*-linked glycosylation sites and three putative glycosaminoglycan sites^15^. As shown in Fig. 3, the apparent molecular mass of podocalyxin is >240 kDa when produced in hiPSCs and hESCs, despite the calculated molecular mass of 55 kDa^10^. These results support the idea that podocalyxin is hyperglycosylated in hPSCs for unknown reasons. In this context, it is worth mentioning that lectins, in general, show relatively low affinity to monovalent sugars with *K*_d_ values in the μM to mM range, whereas they show a greatly enhanced affinity to multivalent glycan ligands, such as mucin-type *O*-glycans, by the so-called “glycoside cluster effect”^16^. Thus, the observed high sensitivity of the GlycoStem test is well explained by the high density of *O*-glycans displayed on the mucinous region of podocalyxin, as illustrated in Fig. 1. This is the reason why a mucin-type *O*-glycan-binding lectin, rABA, as well as other related lectins (i.e., rSRL, rABA and rXCL) performed best as a signal enhancer (Fig. 1)^8^,^11^. In contrast, anti-podocalyxin pAb failed to enhance the rBC2LCN signal. One possibility is that the hyperglycosylation prevented the access of anti-podocalyxin antibody to the protein backbone, in which antigenicity resides. In fact, this explanation agrees with the observation that rABA, which recognizes glycans located at the outermost molecular surface, gave much higher signals to rBC2LCN-captured podocalyxin than anti-podoclayxin pAb in blotting experiments, where protein is denatured.

Previously, we have demonstrated that podocalyxin is a cell surface ligand of rBC2LCN on hiPSCs and hESCs^10^. To confirm whether the detected podocalyxin is soluble, cell culture supernatants were filtrated with 0.22 μm PVDF membrane followed by centrifugation at 21,900 × *g* for 10 min and analyzed by the GlycoStem test. No effect was observed on the signals of the GlycoStem test. Furthermore, ultracentrifugation at 121,492 × *g* for 75 min also gave no effect. Therefore, the detected podocalyxin should be in solution. In this regard, Fernandez et al. reported that podocalyxin is released via exocytic vesicles into the extracellular media both in intact form and as soluble cleaved fragment of ectodomain, when podocalyxin expression vector was transfected into CHO cells^17^. The release of podocalyxin into the extracellular space is in line with the observation of other transmembrane proteins such as CD40L^18^, P-selectin^19^, tumor necrosis factor receptors (TNFRs)^20^, and epidermal growth factor (EGFR)^21^. The soluble podocalyxin might have been cleaved by metalloproteinases, since the protein contains three potential metalloproteinase cleavage sites^17^. Although the functions of soluble as well as transmembrane forms of podocalyxin expressed in hPSCs are largely unknown, it is fascinating to speculate that podocalyxin might regulate the maintenance and morphology of stem cells, similar to the functions proposed in kidney podocytes.

It was recently reported that only a small number of hPSCs is sufficient to produce teratomas^22^. If this is the case, it is absolutely necessary to obtain cell or tissue transplants that are entirely free of tumor-initiating cells^22^. To overcome the tumorigenic risk of hPSCs, several strategies have been proposed including introduction of suicide genes into the cells^23^ and removal of undifferentiated cells from mixed cell populations prior to transplantation^24^,^25^,^26^,^27^,^28^,^29^. However, only minimal attention has been paid to the method to detect and quantify residual hPSCs in differentiated cell populations. In the present work, we have developed a noninvasive method that allows quantitative detection of undifferentiated hPSCs using only a small volume of cell culture supernatant. Future studies should examine the compatibility of the GlycoStem test with specific clinically-relevant differentiation protocols and determine the optimal protocol for each individual case. The developed method provides a novel concept for noninvasive assessment of the safety, properties, and quality of stem cell-based products.

## Methods

### Cell culture

TIG3 hiPSCs (TIG/MKOS #19) were generated as previously described^10^. TIG3 hiPSCs (TIG/MKOS #19) were cultured in DMEM-F12 medium (SIGMA) supplemented with 20% KSR (Invitrogen), 0.05 mM 2-mercaptoethanol (Invitrogen), MEM non-essential amino acids (Invitrogen), Penicillin-Streptomycin (Invitrogen), and 5 ng/mL recombinant human basic FGF (Wako) on mitomycin C-treated SNL feeder cells^10^. 253G1 hiPSCs were cultured in DMEM-F12 medium (Invitrogen) supplemented with 20% of KSR (Invitrogen), 0.1 mM of 2-mercaptoethanol (Sigma–Aldrich), MEM non-essential amino acids (Invitrogen), and 10 ng/ml of recombinant human basic FGF (Wako) on mitomycin C-treated mouse embryo fibroblast feeder cells^30^. 201B7 hiPSCs and 253G4 hiPSCs were cultured in 2.5 mL of StemSure hPSC medium (Wako), ReproFF (ReproCELL), Nutristem (Biological Industries), MEF-conditioned medium (MEF-CM), and mTeSR1 (STEMCELL Technologies) on 6 cm dishes coated with Matrigel (BD Biosciences)^1^,^30^. Human ES cell line, H1, was maintained in 2 mL of mTeSR1 on 6-well plates according to WiCell Feeder Independent Pluripotent Stem Cell Protocols provided by the WiCell Research Institute (www.wicell.org). KhES1 hESCs were cultured on 6-well plate as previously described^31^. After overnight culture, the cell culture supernatants were recovered and centrifuged at 1,400 × *g* for 10 min to remove cell debris. The supernatants were finally stored at −80°C until use. Cells were counted with a hemocytometer or a Vi-CELL Cell Viability Analyzer (Beckman coulter).

### Lectin microarray

rBC2LCN was spotted in triplicates at 1 mg/mL as previously described^12^. Cell culture supernatants (50 μL) of TIG3 hiPSCs (TIG/MKOS #19) (TIG3 hiPSC sup) or control media (Control media) were directly labeled with 100 μg Cy3-NHS ester (GE) and incubated with rBC2LCN immobilized on a glass slide. For the sandwich assay, cell culture supernatants (40 μL) were incubated overnight at 20°C with rBC2LCN-immobilized glass slides at. After washing, Cy3-labeled recombinant lectins (1 μg/mL) were overlayed at 20°C for 3 h. The fluorescence images were acquired using an evanescent-field activated fluorescence scanner (GlycoStation™ Reader 1200, GlycoTechnica Ltd.). The fluorescence signal of each spot was quantified using Array Pro Analyzer ver.4.5 (Media Cybernetics, Bethesda), and the background value was subtracted. The lectin signals of triplicate spots were averaged. Recombinant lectins were prepared as previously described^8^.

### GlycoStem test

Biotin-labeled rBC2LCN (0.1 μg) diluted in PBS (Takara) was immobilized on streptavidin-coated plates (Nunc) at 37°C for 1 h. After washing 5 times with 200 μL of wash buffer (PBS containing 0.1% Triton X-100), 50 μL of cell culture media of hiPSCs and ESCs were allowed to react at 37°C for 1 h. After washing, 50 μL of HRP-labeled rABA (0.1 μg/mL) was overlaid at 37°C for 1 h. After washing, 100 μL of 1-step ULTRA TMB-ELISA (Thermo Fisher Scientific) was then added and developed at room temperature for 30 min. The reaction was stopped by 100 μL of 1 M H_2_SO_4_ and detected at 450 nm. Standard curves were generated using cell culture supernatants of 201B7 hiPSCs cultured in mTeSR1 for 24 h. The apparent cell number was calculated from the linear equation obtained from the standard curve and expressed as AU.

### Western and lectin blotting

Cell culture media (100 μL) were incubated with 10 μL of streptavidin-coated magnetic beads (Life Technologies) immobilized with 1 μg of rBC2LCN at room temperature for 3 h. After washing 5 times with 200 μL of PBST (PBS containing 1% Triton X-100), bound samples were eluted with 20 μL of 0.2% SDS at 95°C for 5 min. The eluted samples were electrophoresed under reducing conditions on 5–20% polyacrylamide gel (DRC). The separated proteins were transferred to a polyvinylidene difluoride (PVDF) membrane and incubated with 0.1 μg/mL of either HRP-labeled recombinant *Agaricus bisporus* lectin (rABA) or goat anti-podocalyxin polycolonal antibody (pAb) (R&D) followed by HRP-labeled donkey anti-goat IgG (x10,000, Jackson ImmunoResearch). Finally, the membranes were developed with Western Lighting Plus (PerkinElmer).

### Flow cytometry

hiPSCs were dissociated with Accutase (Millipore) and resuspended at 2 × 10^5^ cells in 100 μL of MACS buffer (0.5% bovine serum albumin and 2 mM EDTA in PBS), and incubated with anti-SSEA4 (clone MC-813-70, 1:300 dilution, Millipore) followed by AlexaFluor488-labeled anti-mouse IgG (Molecular Probes) or 1 μg/mL of HiLyteFluor 647-labeled rBC2LCN for 1 h at 4°C. Normal mouse IgG (Calbiochem) and HiLyteFluor 647-labeled BSA were used as negative controls, respectively. Cells were stained with propidium iodide (PI) and 10,000 cells were counted using a FACS Aria (BD Biosciences). Data were analyzed with FlowJo software (Tree Star, Inc.).

## Author Contributions

H.T., Y.O., Y.I. designed research. H.T., Y.O., Y.I., K.Hie., Y.A., M.S., K.Hig., M.F., M.W. carried out the experiments, H.T., Y.O., Y.I., M.F., M.W. analyzed data. H.T. and J.H. wrote the paper. S.H., M.A. and J.H. supervised the research.

## Supplementary Material

Supplementary Information Supplementary information
