# Supplementary material for: A medium hyperglycosylated podocalyxin enables noninvasive and quantitative detection of tumorigenic human pluripotent stem cells
Source: Sci Rep. 2014 Feb 12;4:4069. doi: 10.1038/srep04069 (PMC3921628; doi:10.1038/srep04069)
Supplement: Supplementary Information [file srep04069-s1.pdf]

Supplementary information

A medium hyperglycosylated podocalyxin enables noninvasive and quantitative detection of tumorigenic human pluripotent stem cells

Hiroaki Tateno, Yasuko Onuma, Yuzuru Ito, Keiko Hiemori, Yasuhiko Aiki, Madoka Shimizu, Kumiko Higuchi, Masakazu Fukuda, Masaki Warashina, Susumu Honda, Makoto Asashima & Jun Hirabayashi

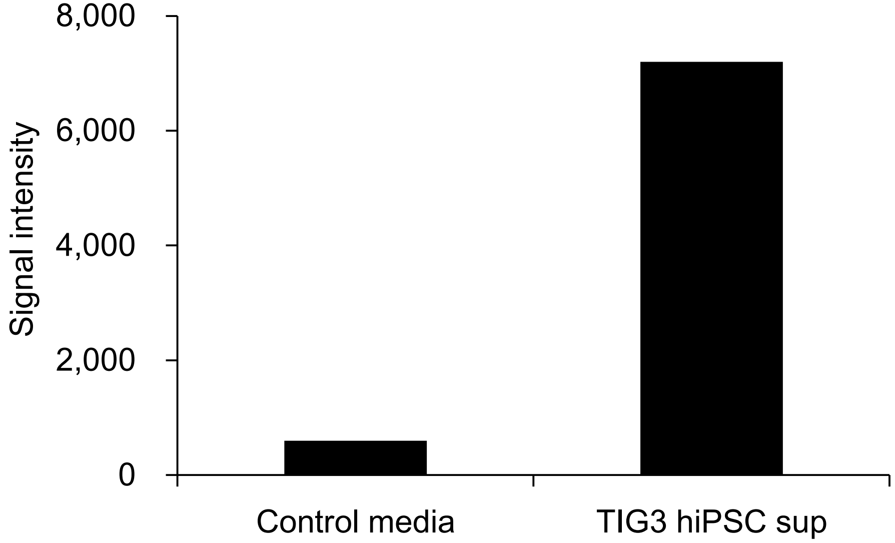

Fig. S1. rBC2LCN binds to cell culture supernatants of hiPSCs. Cell culture supernatants of TIG3 hiPSCs (TIG/MKOS #19, 50 µL) or control cell culture media (Control media, 50 µL) were directly labeled with 100 µg of Cy3-NHS ester. After 10 times dilution with probing solution (25 mM Tris-HCl, pH 7.5, 140 mM NaCl (TBS) containing 2.7 mM KCl, 1 mM $CaCl_2$, 1 mM $MnCl_2$, and 1% Triton X-100), 60 µL of the Cy3-labeled samples were then incubated with rBC2LCN (1 mg/mL) immobilized on a glass slide overnight at 20°C. After washing with probing solution, fluorescent images were acquired using an evanescent-field activated fluorescence scanner, GlycoStation™ Reader 1200 (GlycoTechnica Ltd.) under Cy3 mode. Data were analyzed with the Array Pro analyzer Ver. 4.5 (Media Cybernetics, Inc.). Net intensity value for each spot was determined by signal intensity minus background value. Data are shown as average of triplicate spots.

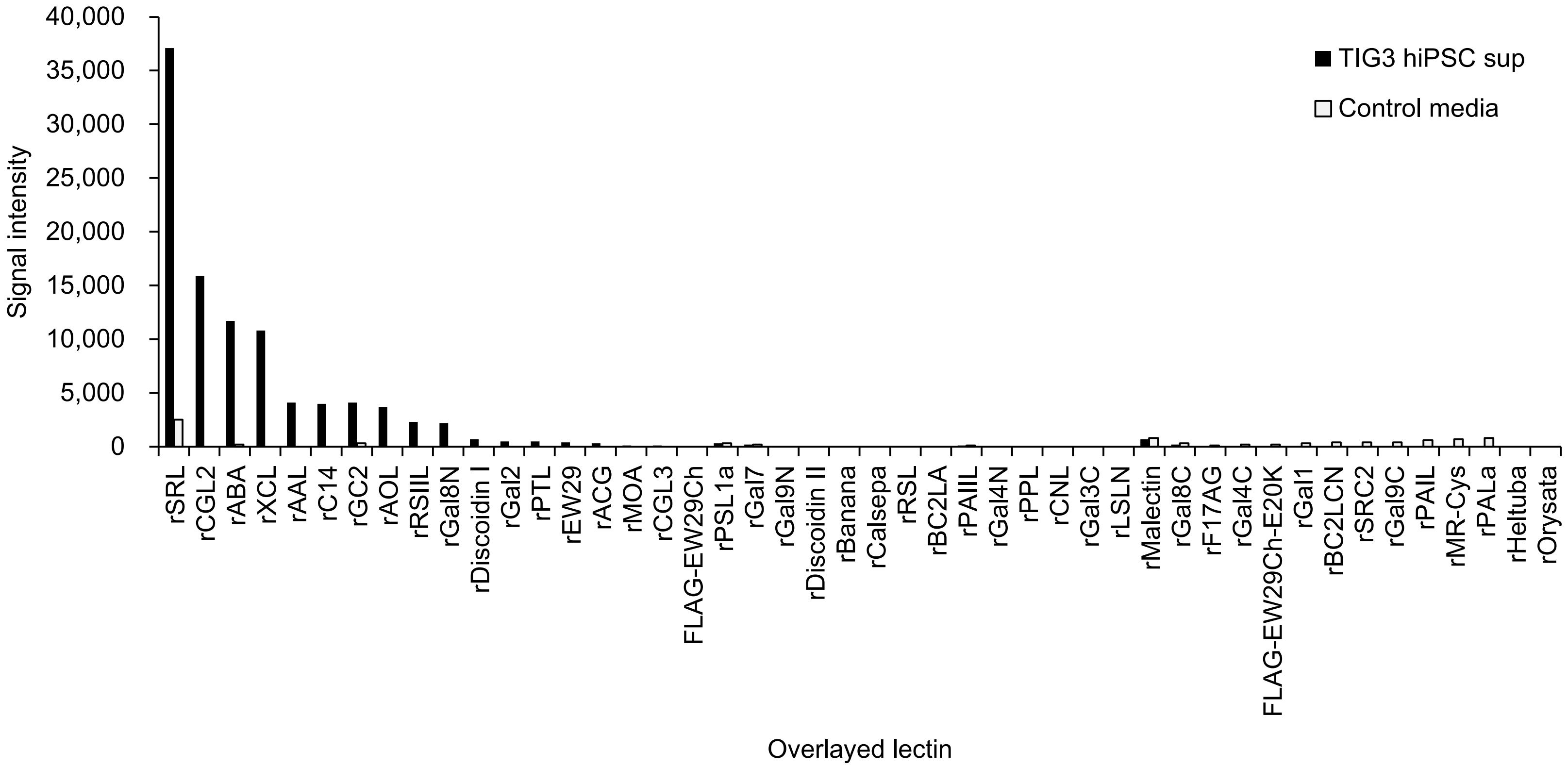

Fig. S2. Screening of overlay recombinant lectin probes. Forty µL of cell culture supernatants of TIG3 hiPSCs (TIG/MKOS #19) (TIG3 hiPSC sup) or control cell culture media (Control media) were incubated with rBC2LCN (1 mg/mL) immobilized on a glass slide overnight at 20°C. After washing with probing solution, Cy3-labeled recombinant lectins (1 µg/mL, 50 µL) were overlayed for 3 h at 20°C and fluorescent images were acquired using an evanescent-field activated fluorescence scanner GlycoStation™ Reader 1200 (GlycoTechnica Ltd.) under Cy3 mode. Data were analyzed with the Array Pro analyzer Ver. 4.5 (Media Cybernetics, Inc.). Net intensity value for each spot was determined by signal intensity minus background value. Data are shown as average of triplicate spots.

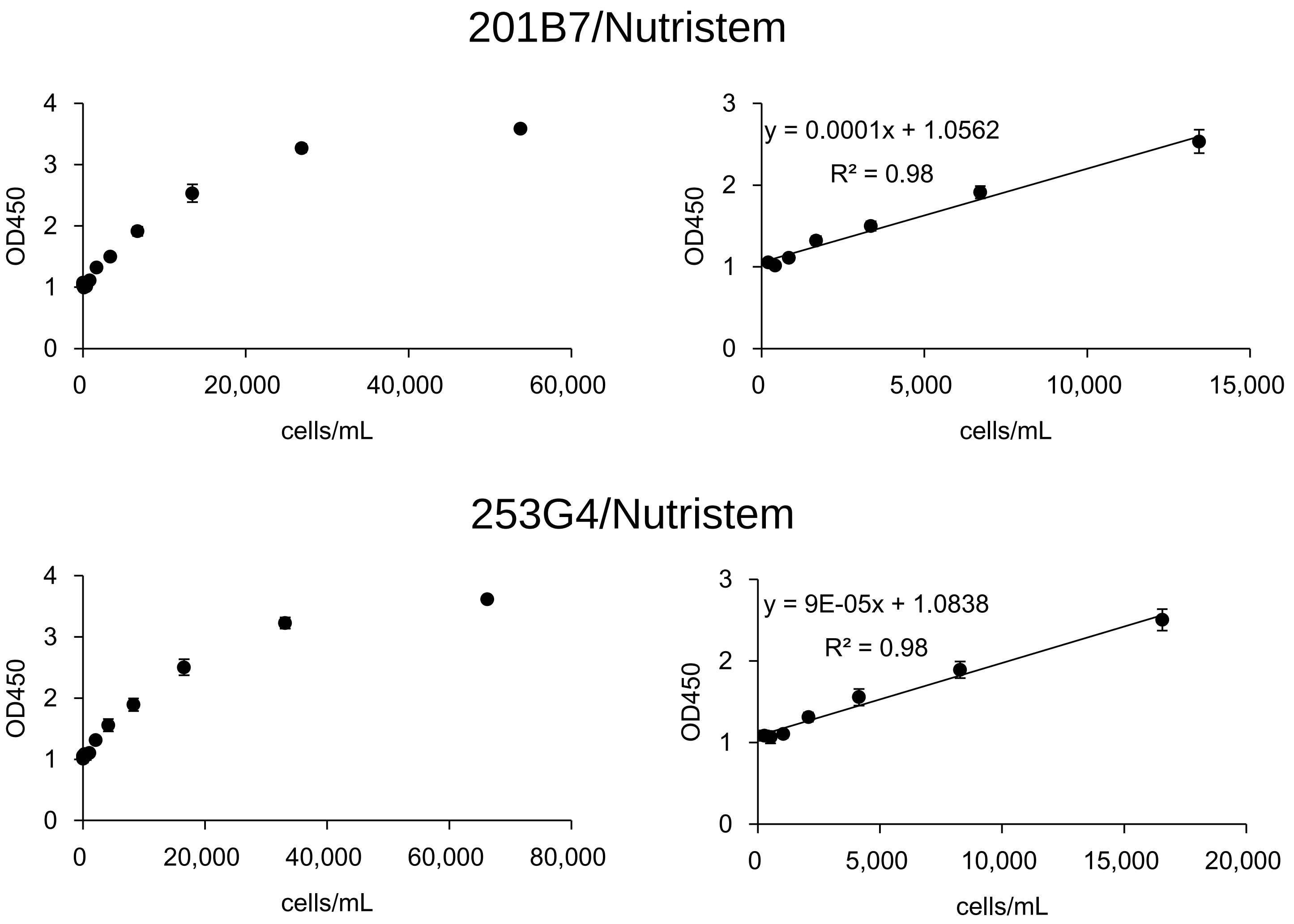

Fig. S3. Standard curve. Cell culture supernatants of 201B7 hiPSCs and 253G4 hiPSCs cultured in Nutristem (Biological Industries) were recovered, serially diluted with Nutristem, while the adhered cells were recovered and counted. The obtained cell culture supernatants were analyzed by GlycoStem test in triplicates. The absorbance at 450 nm of control cell culture media was subtracted from the values obtained from the cell culture supernatants. Data are shown as average ± SD of three independent experiments.

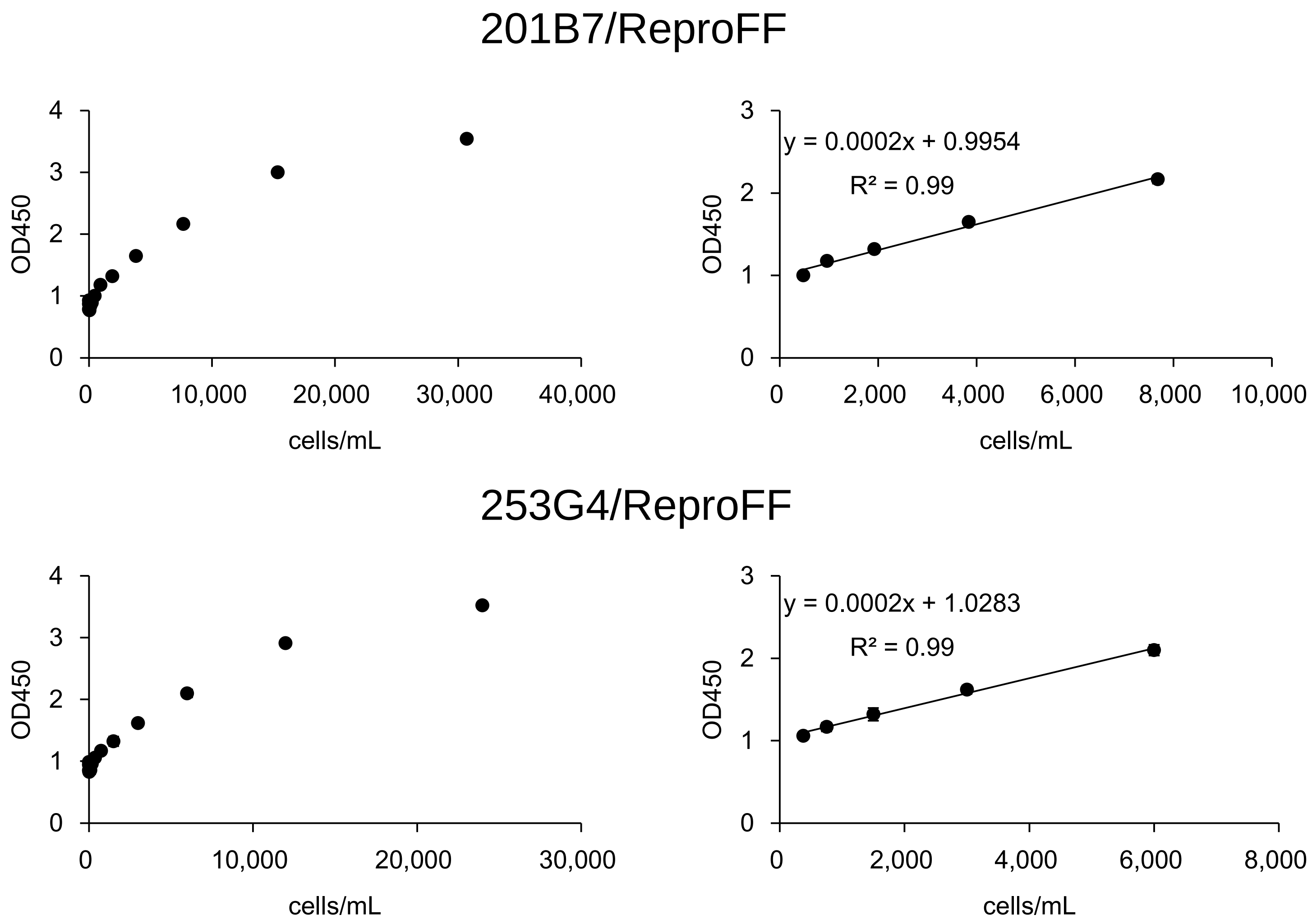

Fig. S4. Standard curve. Cell culture supernatants of 201B7 hiPSCs and 253G4 hiPSCs cultured in ReproFF (ReproCELL) were recovered, serially diluted with ReproFF, while the adhered cells were recovered and counted. The obtained cell culture supernatants were analyzed by GlycoStem test in triplicates. The absorbance at 450 nm of control cell culture media was subtracted from the values obtained from the cell culture supernatants. Data are shown as average ± SD of three independent experiments.

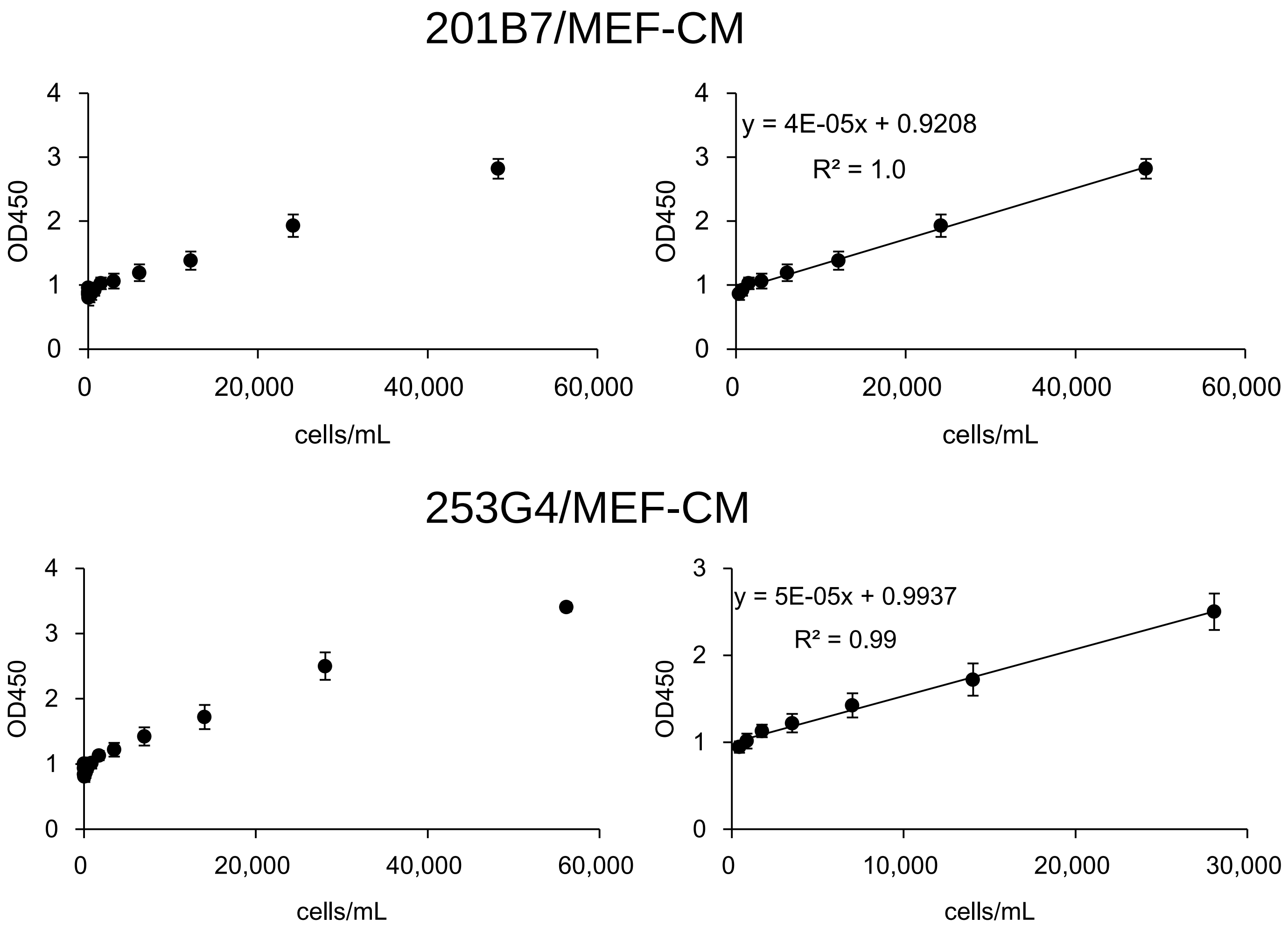

Fig. S5. Standard curve. Cell culture supernatants of 201B7 hiPSCs and 253G4 hiPSCs cultured in MEF-conditioned medium (MEF-CM) were recovered, serially diluted with MEF-CM, while the adhered cells were recovered and counted. The obtained cell culture supernatants were analyzed by GlycoStem test in triplicates. The absorbance at 450 nm of control cell culture media was subtracted from the values obtained from the cell culture supernatants. Data are shown as average ± SD of three independent experiments.

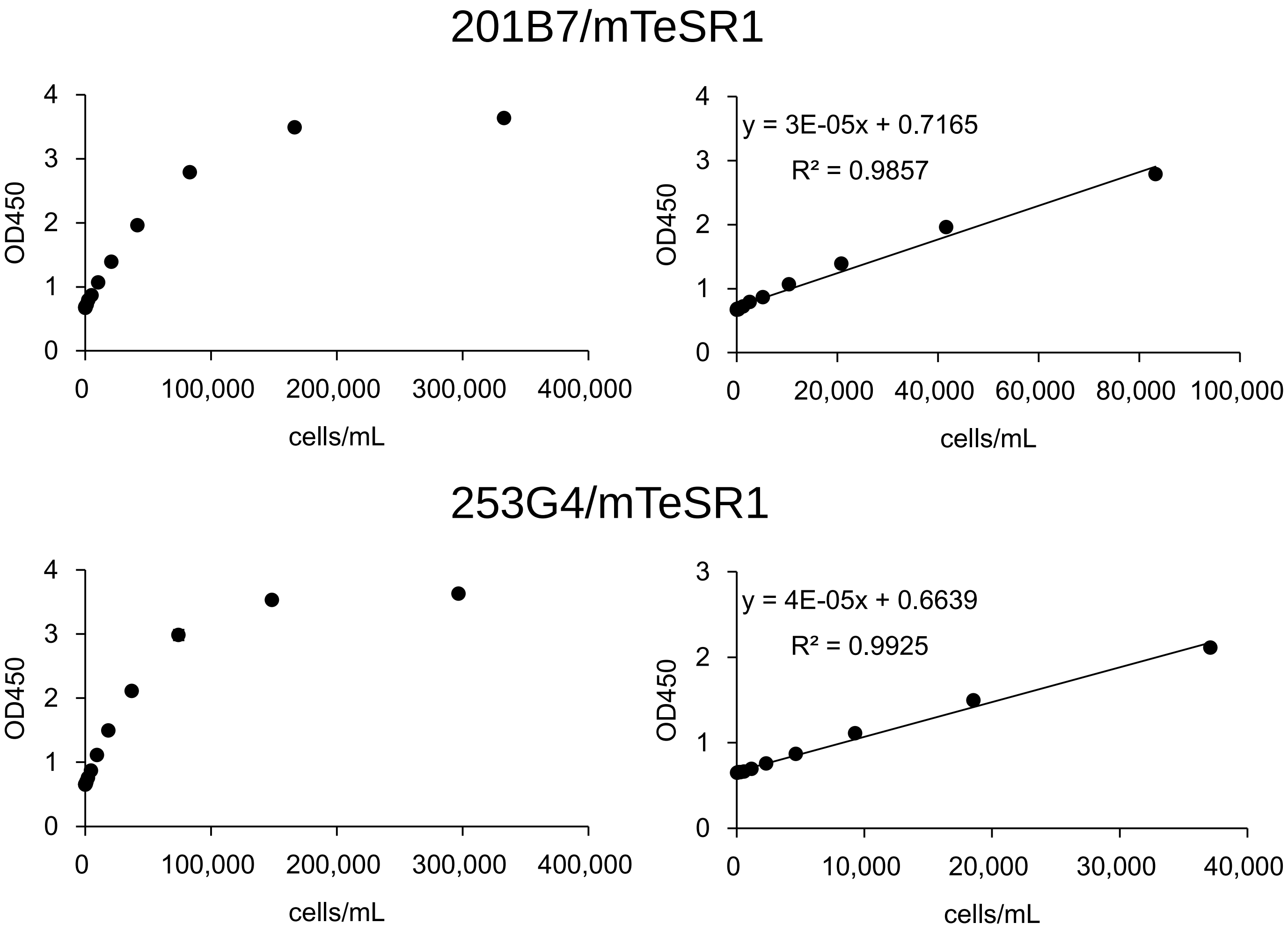

Fig. S6. Standard curve. Cell culture supernatants of 201B7 hiPSCs and 253G4 hiPSCs cultured in mTeSR1 (STEMCELL Technologies) were recovered, serially diluted with mTeSR1, while the adhered cells were recovered and counted. The obtained cell culture supernatants were analyzed by GlycoStem test in triplicates. The absorbance at 450 nm of control cell culture media was subtracted from the values obtained from the cell culture supernatants. Data are shown as average ± SD of three independent experiments.

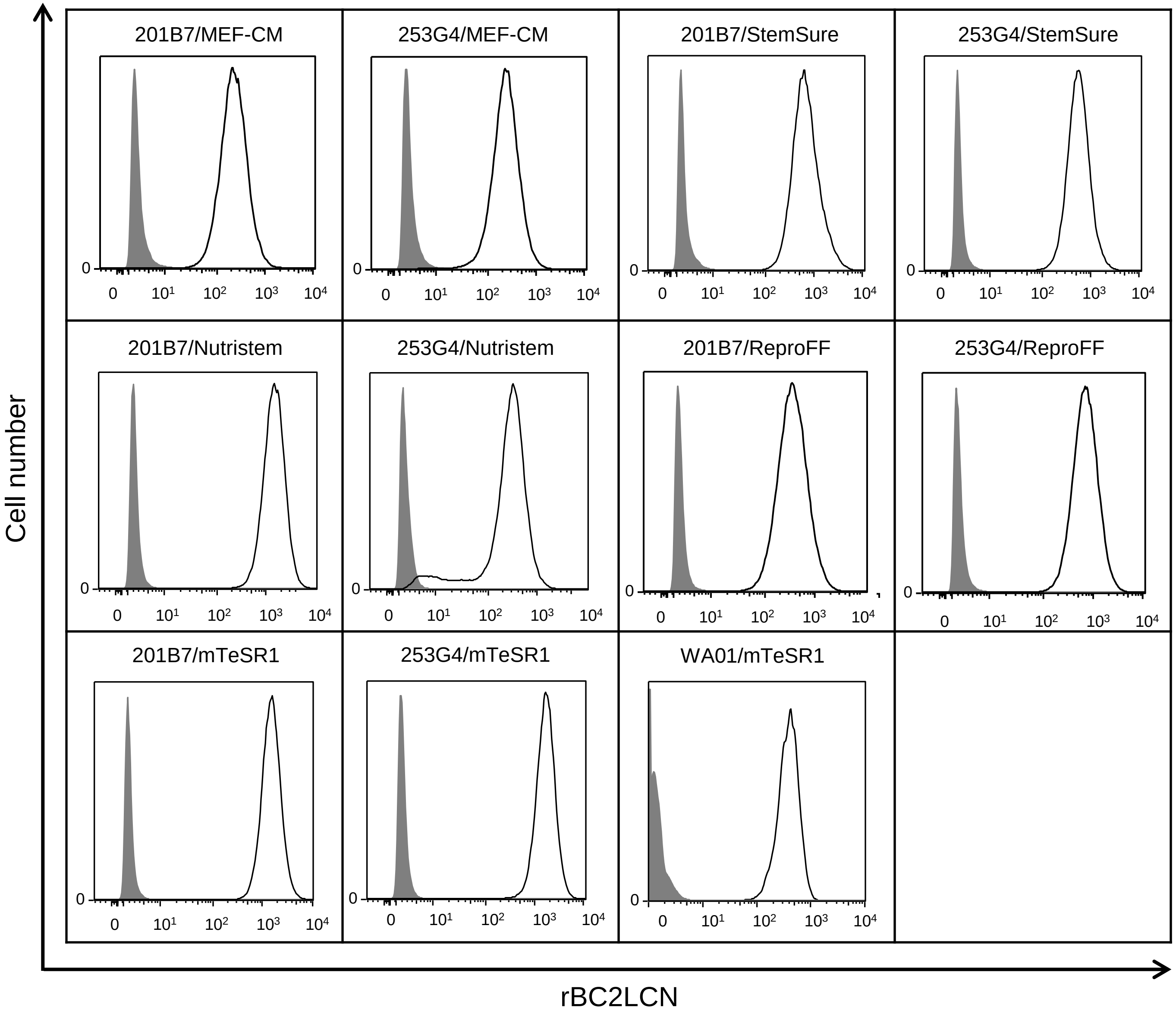

Fig. S7. Staining of 201B7, 253G4, and WA01 by rBC2LCN. Cells were stained with HiLyte Fluor 647-conjugated rBC2LCN and analyzed by flow cytometry. Grey, negative control HiLyteFluor647-conjugated BSA.

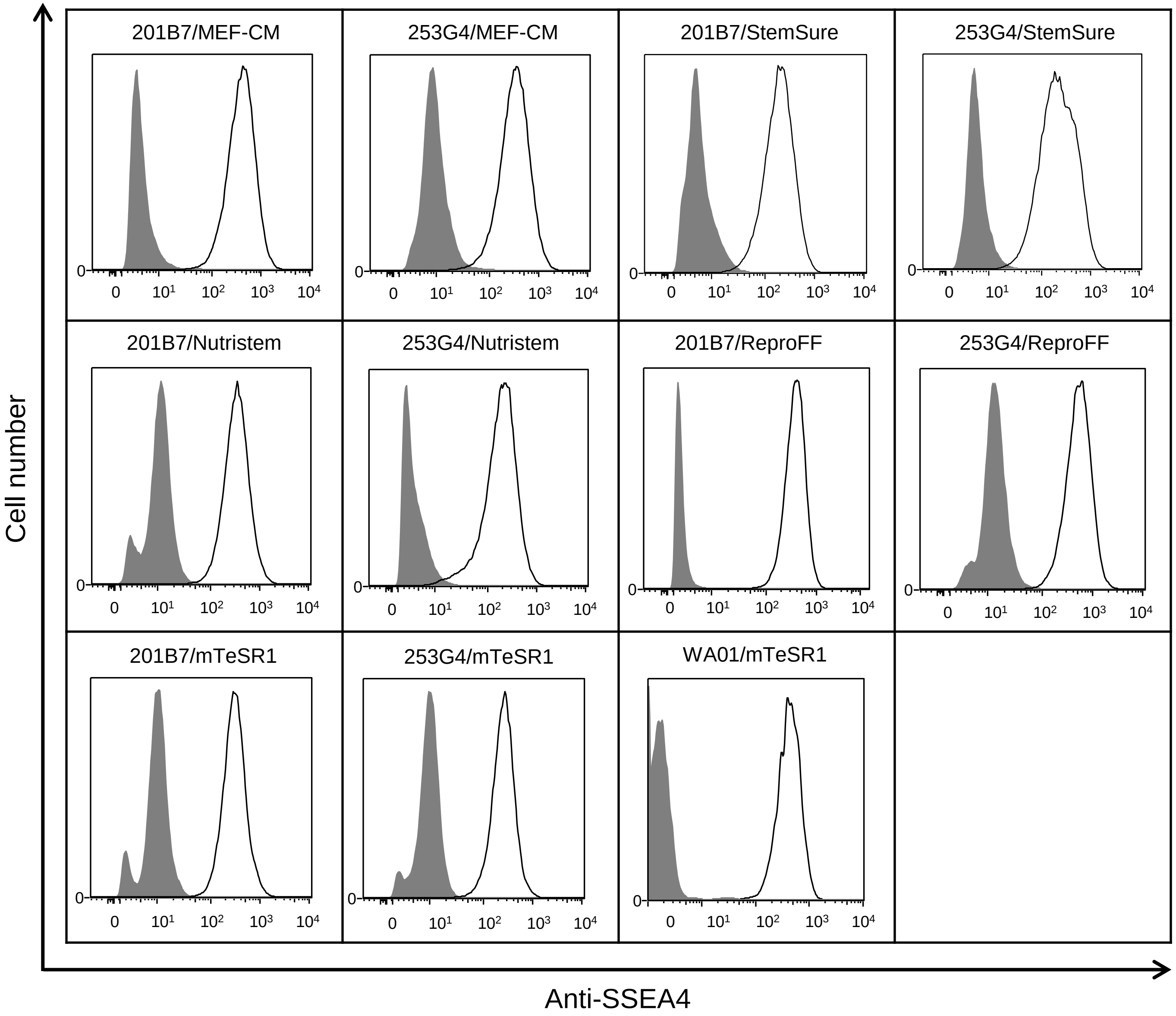

Fig. S8. Staining of 201B7, 253G4, and WA01 by anti-SSEA4. Cells were stained with anti-SSEA4 (clone MC-813-70, 1:300 dilution, Millipore) followed by AlexaFluor488-labeled anti-mouse IgG(Molecular Probes). Grey, isotype negative control IgG.
